# Characterizing the Composition of the Pediatric Gut Microbiome: A Systematic Review

**DOI:** 10.3390/nu12010016

**Published:** 2019-12-19

**Authors:** Kane E. Deering, Amanda Devine, Therese A. O’Sullivan, Johnny Lo, Mary C. Boyce, Claus T. Christophersen

**Affiliations:** 1 School of Medical and Health Sciences, Edith Cowan University, Perth, WA 6027, Australia; a.devine@ecu.edu.au (A.D.); t.osullivan@ecu.edu.au (T.A.O.); c.christophersen@ecu.edu.au (C.T.C.); 2 School of Science, Edith Cowan University, Perth, WA 6027, Australia; j.lo@ecu.edu.au (J.L.); m.boyce@ecu.edu.au (M.C.B.); 3 WA Human Microbiome Collaboration Centre, School of Molecular and Life Sciences, Curtin University, Perth, WA 6102, Australia

**Keywords:** gut microbiome, gut microbiota, gut health, pediatrics, children, diet, short chain fatty acid, SCFA, review

## Abstract

The consortium of trillions of microorganisms that live inside the human gut are integral to health. Little has been done to collate and characterize the microbiome of children. A systematic review was undertaken to address this gap (PROSPERO ID: CRD42018109599). MEDLINE and EMBASE were searched using the keywords: “healthy preadolescent children” and “gut microbiome” to 31 August 2018. Of the 815 journal articles, 42 met the inclusion criteria. The primary outcome was the relative abundance of bacteria at the phylum, family, and genus taxonomic ranks. α-diversity, short chain fatty acid concentrations, diet, *16S rRNA* sequencing region, and geographical location were documented. The preadolescent gut microbiome is dominated at the phylum level by Firmicutes (weighted overall average relative abundance = 51.1%) and Bacteroidetes (36.0%); genus level by *Bacteroides* (16.0%), *Prevotella* (8.69%), *Faecalibacterium* (7.51%), and *Bifidobacterium* (5.47%). Geographic location and *16S rRNA* sequencing region were independently associated with microbial proportions. There was limited consensus between studies that reported α-diversity and short chain fatty acids. Broadly speaking, participants from non-Western locations, who were less likely to follow a Westernized dietary pattern, had higher α-diversity and SCFA concentrations. Confirmatory studies will increase the understanding of the composition and functional capacity of the preadolescent gut microbiome.

## 1. Introduction

The gut microbiota is a complex and dynamic environment containing 10–100 trillion microorganisms represented by 1000 species [1,2,3] involved in numerous biological processes. They assist in the breakdown of foods into metabolically and functionally important metabolites, such as short chain fatty acids (SCFAs) [4] and vitamin K [5]. They also play a role in immune development and several other areas crucial for our future health [6]. For example, a lack of *Bifidobacteria* during infancy may result in the proliferation of pathogenic bacteria or a decrease in the nutritional status of the infant [7]. Later in life, these microorganisms may play a role in the development or mediation of several acute and chronic illnesses [8]. Although there are several studies of the microbiome in ‘normal’ or ‘healthy’ children, they often have small participant numbers and lack comparisons with phenotypic data. Factors, including diet and environment, are significant modulators of the gut but are seldom captured or considered in modelling. This systematic review aims to summarize the literature by characterizing the composition of the gut microbiota of preadolescent children, review the impact of primer selection, and explore associations between diet, geographical location and SCFA concentrations on community structure.

The main development of the microbiome occurs during the first few years of life and may even begin before birth. Until recently, it was believed children were born with a sterile gut. However, emerging evidence suggests otherwise, but newborn gut sterility continues to be controversial [9,10,11,12]. Following birth, many factors can influence the development of the new-born gut microbiome, including method of delivery, exposure to antibiotics, and breastfeeding status [13,14,15,16]. Researchers of one study found that infants received 27.7% of their bacteria from breast milk [15]. During those first few months the most abundant bacteria of the infant microbiome are anaerobic bacteria, such as *Bacteroides, Lactobacillus,* and *Bifidobacterium* [17,18,19]. These bacteria belong to the phyla Bacteroidetes, Firmicutes and Actinobacteria, respectively, and along with Proteobacteria from breast milk, dominate the microbiome during the first year of life [14,20]. Major diversification occurs between the ages of 4–6 months with the introduction of solid foods and continues until the child is 2–3 years old [21]. This coincides with an increase in the relative abundance of butyrate producing bacteria, including bacteria of the families *Ruminococcaceae* and *Lachnospiraceae*, both of the which are from the phylum Firmicutes [22,23,24]. Emergence of bacteria of the phylum Verrucomicrobia also occurs at around two-years old [19]. Overall, findings suggest that a child’s gut microbiome begins to stabilize and form an adult-like composition between two and five years [25,26]. However, this view is not universal, and development of the microbiome may continue until puberty or later [27,28,29]. Environment and diet are considered to be important factors that shape the microbiome over this childhood period.

Geographical region and dietary intake have previously been shown to independently influence the microbial communities, with differences seen between American, Malawian, and Amerindian children and adults [30]. This variation in microbial community was also shown in a study of African and Italian children [31]. These differences may relate to diet; African children consumed a traditional rural African diet (high fiber) whereas European children followed a Western diet (highly processed). In a study of Thai children, conducted by Kisuse et al. [32], those living in an urban environment were more likely to consume a high fat diet, have relatively more bacteria of the genera *Bacteroides* and less *Prevotella* than rural children. Many other studies have documented the acute and continuing influence of diet on the gut microbiome in children and adults [33,34,35,36]. Dietary fiber is particularly important. The three main SCFAs are acetate, butyrate, and propionate, and along with the other SCFAs, are end-products of dietary fiber fermentation by microorganisms in the colon [37]. These metabolites play a positive role in numerous bodily processes, including pH homeostasis, appetite suppression, immunity, and gut health [38,39].

In addition to diet and geography, the method used to assess the gut microbiome can also contribute to differences observed. In studies of the microbiome, hypervariable region selection influences the sequence data and therefore the subsequent microbial community structure. There are nine hypervariable regions of the *16S rRNA* gene [40]. In a comparative analysis of variability within those nine regions of the *16S rRNA* gene, Yang and colleagues [41] concluded that the best representation of the full length gene was the V4-V6 regions, while V2 and V8 were the least reliable regions. In another study that used a well characterized mock community, it was found that compared with V7-V8 and V6-V8 regions, the V4 region showed the highest similarity to the taxonomic distribution of the mock community [42]. In contrast, an older study suggested V1-V3 provide good coverage of the bacterial community [43]. In addition to the variation caused by using different hypervariable regions, longer sequence reads do not necessarily mean more accurate data as shorter reads have less length heterogeneity biases and risk of chimera formation [44].

Previous reviews of the pediatric microbiome have focused on children with a disease or group of diseases, such as inflammatory bowel disease, or have focused on an individual taxon or ratio, such as the Firmicutes: Bacteroidetes ratio. To our knowledge, this will be the first review to look at the pediatric gut microbiome in a healthy population. Furthermore, of the limited research in children, this age group is one that has been frequently overlooked, as noted by Derrien, et al. [45]. This review aims to capture a snapshot of the healthy pediatric gut microbiome using relative abundance data, and to better understand any associations with geographical location and region selected for sequencing and diet. This review will also identify limitations of the current literature and make suggestions for the direction of future research.

## 2. Materials and Methods

### 2.1. Search Strategy

The search strategy, determination of eligibility, extraction, and analysis for this systematic review were all predetermined and included in PROSPERO protocol (ID: CRD42018109599). Preferred Reporting Items for Systematic Reviews and Meta-Analysis (PRISMA) statement guidelines [46] were followed and a Flow Diagram and Checklist are attached as Supplementary Figure S1.

The search strategy aimed to find only published studies. Initially, a limited search of MEDLINE and EMBASE was undertaken on the 20th of September 2018, followed by analysis of the text words contained in the title and abstract. The reference lists of all identified full-text articles were searched for additional studies. Human studies published in English between the 1st January 2000 and the 31st August 2018 were considered for inclusion. Only studies published using next generation sequencing and microarray technologies for bacterial composition identification were considered. Case-control studies and studies of children at predetermined risk of disease were not included in this review. Participants had to be healthy, which was defined as free from any diagnosed disease or illness. Those who were overweight or obese were included, as overweight and obesity are classified as a risk factor rather than a disease [47]. Studies with undernourished or malnourished children were excluded. Keywords were: child * OR pediatric * OR infant OR toddler OR preadolescent OR boy * OR girl * OR prepubescent AND gastrointestinal microbiome OR gastrointestinal microbiota OR gut microbiome OR gut microbiota OR gut health OR gut flora.

### 2.2. Data Extraction

All search results were imported into reference management software Endnote Version 9 (Thomson Reuters). Titles and abstracts were initially reviewed for eligibility. Remaining articles were independently reviewed in full text versions by two of the authors, K.E.D. and C.T.C. Where there was a difference of opinion, consensus was sought before moving to the next study. Studies were included in data extraction phase if they met the following criteria: (1) clinical trial or cohort study; (2) healthy children where the majority of children included in the study were aged 2 to 12 years (determined by mean or median age of the cohort, depending on availability); and (3) employed molecular technologies to characterize part or all of the gut microbiome.

K.E.D. extracted data from eligible studies with assistance from C.T.C. The following demographic variables were extracted: cohort location, cohort name and/or study population, age, sex, sample size, study design, and inclusion and exclusion criteria. Information regarding laboratory techniques included hypervariable region sequenced, sequencing platform, and DNA extraction protocol. The primary outcome variable was relative abundance of taxa at the phylum, family, and genus taxonomic ranks. Data and taxonomic names were extracted as reported. Ideally, they would conform with either the US National Center for Biotechnology Information (NCBI) or List of Prokaryotic names with Standing in Nomenclature (LPSN) naming systems. Taxa that did not fit these criteria was adapted to these norms, where possible. Exploratory, or secondary outcomes, included α-diversity, SCFA concentration, and associations with diet. Data reported in text, figures and tables as ‘unknown’ was either not reported by the author, reported as unknown by the author, or not classified.

For one of the papers identified in the search, the authors decided to group the raw data [28], as significant effort would have been required to untangle multiple time points of microbiome data and the intervention had no significant effect on community structure (unweighted and weighted UniFrac *p* > 0.05). Therefore, there were participants with more than one time point within one age range, potentially weighting the range with their composition.

Additionally, taxa that were classified at different taxonomic ranks were grouped into their higher or lower level taxonomic rank, depending on the analysis. For example, *Asteroleplasma* and uncultured Mollicutes are part of the Tenericutes phylum and were therefore included in this phylum. For genus, where one or more species were listed for an individual genus, the taxa were added and grouped into their respective genus.

SCFA concentrations were converted to μmol/g feces to allow comparisons between studies. Total SCFA concentrations were calculated (referred in-text as calculated total SCFA concentrations) by the summation of acetate, propionate and butyrate concentrations. The ratio of acetate: propionate: butyrate was also calculated.

### 2.3. Statistical Analyses

Relative abundance data were individually weighted according to the sample size of the relevant cohort. Results in tables and figures represent these values. To reduce variance, observations with a weighted average relative abundance of less than 0.05% at phylum rank, 0.5% at family rank, and 0.5% at genus rank were separately grouped into ‘Other’ at their taxonomic rank. Only baseline microbiome data are presented. At the phylum rank, test of the differences in relative abundance between groups (geographic location, age, and 16S region) were conducted in SPSS Version 25, using the Kruskal–Wallis test [48], and Bonferroni correction [49] was applied to minimize Type I error. Significance level was set at 0.05. Firmicutes: Bacteroidetes ratios were calculated using raw abundance data.

Diversity is a measure of number, type and/or evenness of a taxon or group of taxa in an ecosystem, in this case bacteria within the human gut. α-diversity and β-diversity analysis were performed using PRIMER-e Version 7 using extracted data (Quest Research Ltd., Auckland, New Zealand). To calculate α-diversity, data were square root transformed and then Shannon diversity was calculated. The α-diversity values were normally distributed (Shapiro–Wilk test, *p* = 0.535) with no zero values and therefore, a Euclidean distance matrix was created in preparation for PERMANOVA (equivalent to an ANOVA [50]). For β-diversity analysis, missing data were replaced by zeros (required for dissimilarity matrix). To calculate β-diversity, data were square root transformed and a Bray–Curtis dissimilarity matrix produced. PERMANOVAs were run using Type III sum of squares and 99,999 permutations [50]. Where less than 500 unique permutations were completed, a Monte Carlo simulation was utilized in the determination of the *p*-values.

## 3. Results

A total of 42 studies, which included more than 2000 participants, were included (Table 1). Relative abundance data were not available for 14 of 42 studies [21,30,51,52,53,54,55,56,57,58,59,60,61,62]. Most studies were from Asia, Europe, and North America, with 12 studies each. The average age of participants in each study ranged from 2.0 to 11.3 years. Of the 42 studies identified, 18 (42.9%) reported phylum rank data, 10 (23.8%) reported family rank data, and 19 (45.2%) reported genus rank data. Of the phylum rank studies, the most sequenced hypervariable region was the V6 region (n = 7, 38.9%). Only one study sequenced the whole *16S rRNA* gene (5.56%). The review identified 13 phyla, 72 familiae, and 200 genera.

### 3.1. Phylum Level Impact of Geographical Location, Age and 16S rRNA Region

**Figure 1:**
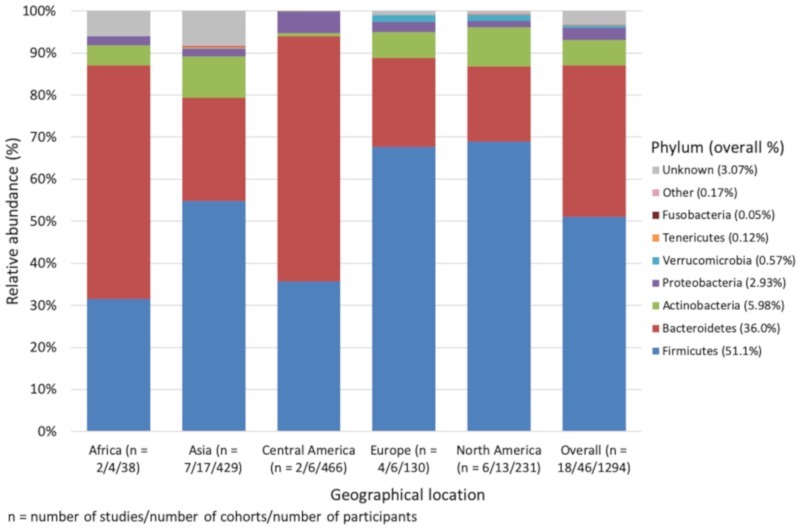
Weighted mean relative abundance (%) of bacteria by geographical location at the phylum level. Other includes: Cyanobacteria, Lentisphaerae, Spirochaetes, Elusimicrobia, Synergistetes, Euryarchaeota. Unknown refers to bacteria that were either not reported, unknown or unclassified.

Overall, at the phylum taxonomic rank, the microbiome was dominated by Firmicutes (weighted overall average relative abundance = 51.1%), Bacteroidetes (36.0%), Actinobacteria (5.98%) and Proteobacteria (2.93%). In addition to these major phyla, Verrucomicrobia (0.57%), Tenericutes (0.12%), Fusobacteria (0.05%) and an unclassified portion (3.07%) were also detected (Table S1 and Figure 1). There were lower proportions of Firmicutes in African (31.6%) and Central American children (35.7%) compared to Western regions (Europe: 67.7% and North America: 69.0%). Firmicutes and Bacteroidetes in European and Central American children were significantly different (*p* = 0.041, and *p* = 0.038, respectively). In European and North American children, the Firmicutes: Bacteroidetes ratio was greater than African and Central American children (3.21 and 3.88, respectively, compared to 0.57 and 0.61, respectively), with Asian children in between (Firmicutes: Bacteroidetes ratio of 2.23). Proportions of Actinobacteria were significantly higher in Asian than Central American children (*p* = 0.035). Note there are only a small number of African children (2.9%) compared to the other four populations. Regarding diversity, Central American children had significantly lower α-diversity compared to the other groups (North America *p* = 0.003, Asia *p* = 0.004, Africa *p*(Monte Carlo) = 0.033, Europe *p*(Monte Carlo) = 0.015). For β-diversity there were similar findings, with Central American children being significantly different to the other four groups. Africa also reported significantly different β-diversity to the other four groups.

**Figure 2:**
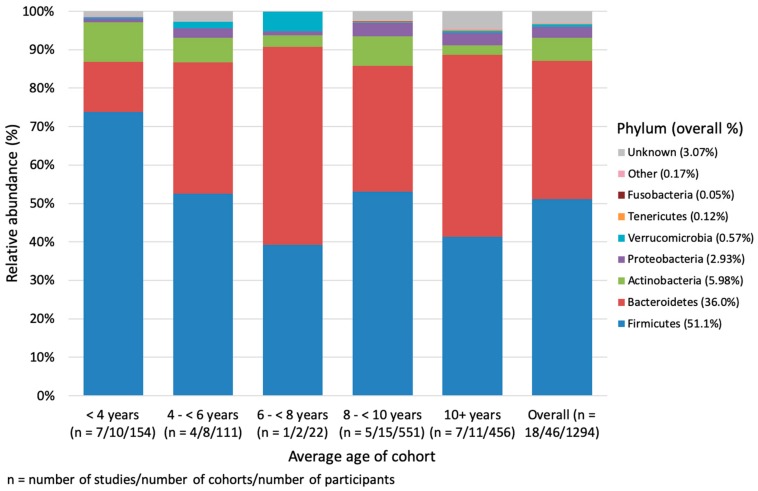
Weighted mean relative abundance (%) of bacteria by ascending average age of the cohort at the phylum level. Other includes: Cyanobacteria, Lentisphaerae, Spirochaetes, Elusimicrobia, Synergistetes, Euryarchaeota. Unknown refers to bacteria that were either not reported, unknown or unclassified.

Within age ranges, the gut microbiome was initially dominated by Firmicutes (73.8%, compared to Bacteroidetes 13.0%) in children under 4 years old. In the following years, the relative abundances of the two major phyla stabilized at comparable proportions (Table S2, Figure 2). A general decrease in Actinobacteria was observed as children age, which is offset by a general increase in Proteobacteria and not reported, unknown or unclassified bacteria. There were no significant differences in relative abundances, α-diversity or β-diversity between the age groups.

**Figure 3:**
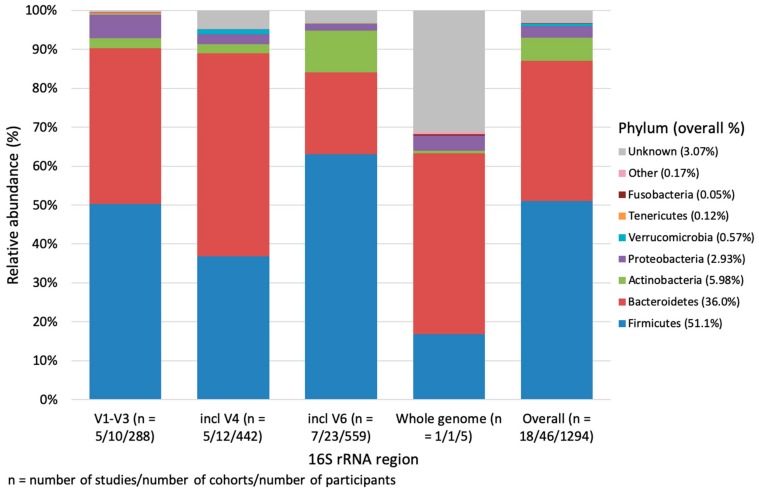
Weighted mean relative abundance (%) of bacteria by *16S rRNA* region at the phylum level. Other includes: Cyanobacteria, Lentisphaerae, Spirochaetes, Elusimicrobia, Synergistetes, Euryarchaeota. Unknown refers to bacteria that were either not reported, unknown or unclassified.

How the microbial composition is detected, metagenomics or *16S rRNA* amplicon, also impacts the outcome. Of the 1294 participants (18 studies) that had phylum rank data, less than 1% had whole genome data available (Table S3, Figure 3). Seven of the 18 studies sequenced regions that included the V6 region whereas V4 and V1-3 was represented by five studies each. V6 region studies had significantly higher proportions of Firmicutes and Actinobacteria than those who sequenced the V4 region (*p* = 0.004 and *p* = 0.001, respectively). V1-V3 region studies had significantly higher proportions of Proteobacteria than V4 and V6 region studies region (*p* < 0.001 and *p* = 0.007, respectively). α-diversity was not significantly different (*p* = 0.355) by region. Whereas, β-diversity analysis indicated that V6 region studies were significantly different to V1-V3 region studies (*p* = 0.009), V4 region (*p* = 0.007) and whole genome data (*p* = 0.043). Whole genome was also significantly different to the other two variable regions (V4 region *p* (Monte Carlo) = 0.041 and V6 region *p* (Monte Carlo) = 0.016).

### 3.2. Family Level Impact of Geographical Location, Age and 16S rRNA Region

At the family level, the gut was dominated by *Bacteroidaceae* (17.5%), *Lachnospiraceae* (16.8%), *Ruminococcaceae* (13.9%), *Prevotellaceae* (12.1%) and *Bifidobacteriaceae* (5.09%). These taxa, along with the seven others presented in the family tables (Tables S4–S6 and Figures S2–S4) represent almost 95.0% of the classified bacteria. Due to the small numbers of studies reporting family rank data (n = 10), results from this section should be interpreted with caution.

Within the geographical location analysis, the proportion of *Prevotellaceae* in African children (46.5%) was almost four times higher than the average of 12.1% (Table S4 and Figure S2). It is important to note there is only one African study with a small number of participants. Asian children had relatively high proportions of *Bifidobacteriaceae* (12.0%) and *Peptostreptococcaceae* (1.96%) compared to the overall averages (5.09% and 0.76%, respectively). Conversely, European children reported higher proportions of *Ruminococcaceae* (27.8% compared to average of 13.9%), and lower proportions of *Prevotellaceae* than the group average (1.96% compared to 12.1%).

The majority of the participants were from cohorts with a mean age of 8–10 years or 10+ year older (90.9%) (Table S5 and Figure S3). There were no studies in the 6–8 years old range. The youngest group (<4 years) had the greatest proportion of *Bacteroidaceae* (31.05%) and least amount of *Prevotellaceae* (2.93%). In the 4–6 years old group, taking note of the relatively small proportion of participants in this age range (3.20%), *Prevotellaceae* was almost three times higher than the overall weighted average of the groups (33.4% compared to 12.1%). In the 8–10 year old group, proportions of *Bifidobacteriaceae* were five-fold higher than the 10+ years group. *Ruminococcaceae* relative abundance was comparable in the three age groups that reported this taxon.

Within the *16S rRNA* region data (Table S6 and Figure S4), both V1-V3 region and whole genome studies had relatively high proportions of not reported, unknown or unclassified bacteria (84.0% and 66.3%, respectively). *Enterobacteriaceae* proportions in the V1-V3 study were five times higher than their closest group (4.07% compared to 0.81% in V6 region cohorts). Studies sequencing the V4 region had low unknown bacteria (3.82%) compared to the other groups. V4 region studies were characterized by relatively higher abundances of *Bacteroidaceae, Prevotellaceae, Alicaligenaceae* and *Rikenellaceae* than overall populations. In comparison, V6 region studies were characterized by higher proportions of *Lachnospiraceae, Bifidobacteriaceae, Coriobacteriaceae* and *Peptostreptococcaceae* (Table S6).

### 3.3. Genus Level Impact of Geographical Location, Age and 16S rRNA Region

At the genus taxonomic rank, the most dominant bacteria were *Bacteroides* (16.0%), *Prevotella* (8.69%), *Faecalibacterium* (7.51%), *Bifidobacterium* (5.47%) and unclassified *Lachnospiraceae* (3.26%). These taxa, along with the 19 others (Tables S7–S9), accounted for 89.0% of all classified bacteria. Overall, 29.8% of bacteria were not reported, unknown or unclassified. This was higher than the proportion of unclassified bacteria at the family rank (23.4%) and more than nine times higher than the rate at the phylum level (3.07%).

In African children (0.99% of participants), *Prevotella* dominated the gut (53.0%) and was higher than any other population (Table S7 and Figure S5). Central American children were characterized by the greatest relative proportions of *Bacteroides* (23.1%) as well as *Prevotella* (14.2%), unclassified *Lachnospiraceae* (4.28%) and unclassified *Ruminococcaceae* (3.59%). Central American children (0.40%) had low proportions of *Bifidobacterium* compared to the rest of the populations (ranged from 5.69–9.71%). *Bifidobacterium* was not reported in the one African study. *Faecalibacterium* was comparable between all populations (ranged from 4.0–9.0%, average = 7.5%).

Independent of the spike in the average weighted relative abundance of *Bacteroides* in cohorts aged 4–6 years, there was a general increase in relative abundance with age (Table S8 and Figure S6). This trend was similar for *Prevotella* and *Dialister*. It was reversed for *Bifidobacterium, Ruminococcus* and *Streptococcus*. In young children (<4 years), *Lachnospiraceae* (9.77%), *Bifidobacterium* (8.26%), unclassified *Ruminococcaceae* (6.06%), *Clostridium XIVa* (5.74%) and *Clostridium* (4.86%) characterized the gut. However, children >10 years had relatively higher *Bacteroides* (20.3%), *Prevotella* (10.99%), unclassified *Ruminococcaceae* (3.79%) and *Dialister* (1.50%).

In congruence with family rank data, there were large proportions of not reported, unclassified or unknown bacteria in the V1-V3 region data (67.9%) (Table S9 and Figure S7). Note that studies rarely reported data at every taxonomic level and that is why there are differences in proportions of unknown or unclassified data at family and genus ranks. Studies including the V4 region were dominated by *Bacteroides* (27.1%), *Prevotella* (11.2%) and unclassified *Lachnospiraceae* (7.12%). In V6 region studies, *Bifidobacterium* was the most relatively abundant bacteria (11.5%), followed by *Faecalibacterium* (9.59%), *Bacteroides* (9.04%) and *Prevotella* (7.97%). Two whole genome studies followed a similar pattern to the V6 region studies, however, participants in this group had higher proportions of *Alistipes* (8.15%), *Eubacterium* (6.84%) and *Dialister* (3.28%) compared to the overall average (0.61%, 0.93% and 0.84%, respectively). *Blautia* proportions are several times higher when the V6 region was analyzed (6.03%) than all other groups.

### 3.4. α-Diversity as Reported by the Included Studies

Of the 42 studies, α-diversity was reported in 26 (61.9%) and five key measures were identified; number of observed operational taxonomic units or species (S), Shannon Diversity (H’), Simpson Diversity (λ), Inverse Simpson (1/λ), Species Richness (Chao1), and Phylogenetic Diversity (PD) Whole Tree (Table S10). The most common α-diversity metric was Shannon Diversity (n = 17 studies), and results ranged from 2.21 in 4–8-year-old North American children to 6.90 in 7–9-year-old Asian children. Data may be transformed and/or normalized prior to diversity calculations, making between study comparisons limited. However, comparisons within studies are still valid. In one study, age was associated with significantly different Shannon Diversity and Inverse Simpson Diversity (although not linear but u-shaped with age) [66]. However, in a follow up study using the same participants, age was not associated with α-diversity [28]. In the comparison of geographically different populations, three of the four studies reported significantly greater diversity in the more rural or less developed populations [31,32,72]. The fourth study showed an increasing trend as the population became more rural, however, it was not significant [67].

### 3.5. Comparison of SCFA Concentrations

Nine studies reported SCFA concentrations (21.4%) (Table S11). Large variations in SCFA concentrations were observed. For example, calculated average total SCFA concentrations in westernized populations ranged from 29.6 μmol/g [31] to 188.4 μmol/g [64]. There was also some variation in the acetate:propionate: butyrate ratios reported for westernized population studies (2.7:0.9:1 to 8.3:2.5:1). Several of the studies reported quite different analytical methods for the analysis of the SCFA. For example, De Filippo et al. [31,67] took advantage of the volatile nature of the SCFA and used solid phase micro-extraction to extract the SCFA from headspace, followed by gas chromatography mass spectrometry (GC–MS) to determine their concentrations. The use of isotopically labelled SCFA as internal standards and a highly specific detector, mass spectrometry helped reduce potential matrix effects and compensate for analyte loss during sample preparation/extraction respectively. Keonig et al. [25] also used GC-MS and isotopically labelled SCFA as internal standards, but extracted the SCFA into organic solvent, and chemically derivatized them before direct injection onto the GC. Other studies used liquid chromatographic [62,84] and capillary electrophoretic methods [32,77]. Payne et al. [62] provided no details with respect to detection or the use of internal standards. Murugesan et al. [84] using high performance liquid chromatography and Riva et al. [77] using capillary electrophoresis both used a non-selective UV detector. UV detection is problematic, particularly at low wavelengths, as coeluting interferences can contribute to the signal. Given the range of extraction and detection methods used across the nine studies, comparisons across the studies is problematic. In addition, two of the nine studies reported SCFA in millimolar; which are not comparable to the units, umol/g, used in the other studies. Despite the variation in concentrations, there were observable trends within studies. African children reported significantly higher concentrations of total SCFA, acetate, propionate, butyrate, and valerate than European children [31]. This association was explored further by De Filippo et al. [67], which showed rural African populations had greater SCFA concentrations than urban African populations. This difference in SCFA concentrations between rural and urban environments was also reported for a Thai study of 45 children [32]. The Thai study also reported the rural children had significantly higher butyrate concentrations (*p* < 0.05), but age and gender were not predictors of SCFA concentrations [32].

### 3.6. Dietary Analysis

Less than half of the identified studies collected any dietary intake data (Table S12). Therefore, results reported here are general observations from individual studies. Bacteroidetes was positively associated with servings of fruit/day [64] but negatively with fat intake as a proportion of total energy [75]. In contrast, Firmicutes was positively correlated with fat intake [75]. At the genus level, fat intake ratio was negatively associated with *Prevotella*, *Succinivibrio,* and *Catenibacterium* and positively associated with *Bacteroides*, *Ruminococcus,* and *Blautia* [75]. *Bacteroides* was also positively correlated with servings of fruit/day [64]. Regarding measures of microbial diversity, servings of fruit/day and intake of refined carbohydrates were negatively correlated with Chao Index and refined carbohydrates was also negatively associated with PD whole tree [64]. Dairy serve intake was negatively associated with Shannon Diversity and Chao Index [80]. Diet explains between 7% and 13% of observed microbial variation [75,80].

## 4. Discussion

### 4.1. Overall Findings

Overall, the pediatric gut microbiome was characterized by high proportions of Firmicutes, Bacteroidetes, Actinobacteria and Proteobacteria at the phylum rank. These were supported by minor phyla such as Verrucomicrobia, Tenericutes and Fusobacteria. At the family rank, the dominant bacteria included *Bacteroidaceae, Lachnospiraceae, Ruminococcaceae, Prevotellaceae* and *Bifidobacteriaceae.* At the genus taxonomic rank, the dominant bacteria were *Bacteroides*, *Prevotella*, *Faecalibacterium* and *Bifidobacterium*. Overall, there is good agreement between the dominant bacteria at the phylum, family and genus ranks. Based on the data collated for this review, geographic location and 16S RNA region sequenced were independent factors of community structure, while age was not.

### 4.2. 16S rRNA Sequencing Region and the Microbiome

Further investigation of β-diversity results suggests that the differences between cohorts are more complicated than the three factors discussed. For example, of those who sequenced V1-V3 hypervariable regions, there was no significant difference in β-diversity by geographical location. In contrast, all three geographical locations (Europe, North America, and Central America) were significantly different to one another in studies that sequenced the V4 hypervariable region. Other studies have independently identified geographical location as a factor in community structure, one sequencing the V4 region [30] and the other the V1-V2 hypervariable region [86]. Children from Western geographical areas had a similar microbiome structure at the phylum level. Both North American and European children had high proportions of Firmicutes compared to the other geographical regions. This similarity was also seen at the family rank, albeit in a less distinct way. The contrasts extend to examining hypervariable region sequenced within geographical location. For example, within North American studies, all hypervariable regions were significantly different to one another but within Asian studies, the only significant difference in β-diversity was between V6 region studies and whole genome studies (V4 region studies not present). As discussed, certain hypervariable regions, in particular the V4 region, may provide more accurate representation of the true community [41,42]. These findings further indicate that differences in study design influence community composition and limit inter-study comparisons.

### 4.3. Diet, Geographical Location, SCFA, and the Microbiome

In addition to geographical location, other factors such as diet are important when considering the composition and modulation of the microbiome. For example, the community composition of children from Asian children reporting in Nakayama et al. [74], who were mainly from urban environments, was similar to that of European and North American children reported in this review. This was observed in the 8–10 year old category, where the majority of participants are from Asian countries, with high proportions of Firmicutes. This is likely to be independent of sequencing region as multiple regions were covered. It may be reflective of urban Asian populations transitioning from a traditional plant-based diet to a more Westernized diet [87]. Lower fat and sugar consumption in the rural compared to urban environments has been noted [32]. This transition is also reflected in the Firmicutes: Bacteroidetes ratio, where children from Western regions had higher ratios than African and Central American children. Asian children had a Firmicutes: Bacteroidetes ratio that was between Western and African regions. Differences were also observed between Thai children: children from an urban setting had a low ratio (0.624), while those in the rural group had a higher ratio (0.856), despite the rural children consuming significantly more vegetables and rice and there being no significant difference in overall energy intake [32]. These reported differences also depended on *16S rRNA* region as the ratio was lower in those who sequenced V1-V3 region compared to the other hypervariable regions. Several authors have proposed that this ratio is associated with obesity status [88], with those having a higher ratio being more likely to be overweight or obese according to animal studies [89], but the evidence is inconclusive in human studies [90,91]. As our analysis indicate this ratio can merely be a reflection of the *16S rRNA* region sequenced so caution has to be taken when comparing across studies.

Despite fewer studies from less developed regions, α-diversity tended to be higher in African and Central American children [92]. Rural status may not be the only geographical factor. Significantly different α-diversity was seen in a study of 10 Asian cities, within five countries [74]. α-diversity has been considered a proxy for health status with higher diversity being preferable [91], however, higher α-diversity is not always associated with positive health status [93]. As a summary metric, α-diversity does not take into consideration which bacteria are present, only the amount or distribution of the total bacteria. Yatsunenko and colleagues [30] proposed that α-diversity increases over the lifespan, yet this research suggests, at least in children, there is no such relationship.

The high proportions of Bacteroidetes in African and Central American children (Figure S5) are the result of having more bacteria capable of fermenting fiber than Western populations namely *Prevotella*. Bacteria within this genus are recognized for their ability to ferment fiber to produce SCFAs [94] and are more abundant in populations who follow a traditional African high-fiber plant-based diet [31,67]. Despite the higher SCFA concentrations in African children when compared to western populations within a study, there was a large variation between studies. For example, there was a five-fold difference between the lowest and highest total calculated SCFAs in western populations. Although the shortcomings in the study methodologies could not explain such a wide variation, concentrations of SCFAs may be influenced by several factors, including volatility of the sample and potential loss of analytes, extraction technique and platform used for analysis [95]. There was also variation in the SCFA ratios, which typically exist in a 3:1:1 ratio in the gut [38], however these were closer to expectation and may be better representations of the metabolic activity. For this reason, SCFA ratios should be considered in future research, along with standardized analysis techniques and factors outside those explored in this study (age and geography), such as diet [39,67,94,96,97].

### 4.4. Association Between Age and the Microbiome

Age was not associated with changes in community structure, although there were trends in specific taxa. Actinobacteria generally decreased with age and Proteobacteria increased. *Bifidobacterium*, a dominant genus that sits within Actinobacteria, gave a similar trend, decreasing with age. Bifidobacteria is one of the early colonizers of the newborn microbiome and is involved in the breakdown of non-digestible carbohydrates [98]. It has been shown to be associated with several positive health outcomes [99], including the prevention or treatment of cancer in animal models [100,101] and the reduction of diarrhea episodes in infants [102,103]. Despite the quantity of research in the area, the researchers identified no obvious reason for the decrease in relative proportions of Bifidobacteria from early childhood into puberty and may be inversely related to proliferation of other bacteria, such as Proteobacteria. This phylum contains some of the most well know pathogens, including the genera *Escherichia*, *Salmonella*, *Vibrio,* and *Helicobacter* [104] and bacteria that may or may not be beneficial such as *Sutterella* [105,106]. As noted by Derrien et al. [45], there is a paucity of research in pre-school (3–6 years) and primary age (6–12 years) children. However, the results from this review are in line with other research that suggests there are limited changes in α- and β-diversity after the early years of life and the microbiome typically resembles an adult-like composition [14,45,107].

### 4.5. Limitations of the Current Research

One of the challenges of next generation sequencing research is producing data that accurately represents the microbial community. This current review found that the selection of sequencing region influences the community structure observed in children, which aligns with other research that have examined the nine hypervariable regions [41]. Other factors can affect data prior to statistical analysis thus rigorous and repeatable methodology, and choice of reference database is important to ensure robust generalizable results. A comparison of data processing workflows found that although diversity and relative abundances were different, the biological conclusions were similar, suggesting generalizability of results [108]. A number of comparative studies have shown that bacteria of lower relative abundance, which are as biologically important, are more likely to be classified differently and the potential importance should not be discounted [109,110]. There are also a number of other factors that influence microbiome data, including samples collection design, DNA extraction protocols and specific workflow decisions, which have led to calls for developing standard workflow practices [111,112,113,114,115].

Although beyond the scope of this review, an influential factor in taxonomic classification is the database used. Advances in the field mean that recently published studies classified data with updated databases, limiting direct comparisons between the studies. For example, several bacteria were reclassified from *Clostridium Cluster XIVa* to a new genus, *Blautia*, in the late 2000s [116]. Similarly, the genus, *Xylanibacter*, represented 20% of the ‘Other’ bacteria in De Filippo et al. [31]. The genus is no longer listed in the NCBI database or Genome Taxonomy Database (GTDB). It is still listed by the List of Prokaryotic Names (LPSN), however, the one species listed there, *Xylanibacter oryzae*, was reclassified to the genus *Prevotella* as *Prevotella oryzae* in 2012 [117]. As the ability to isolate and identify bacteria improves, and methods standardize, the ability to compare between studies should improve. A substantial proportion of studies developed their own taxonomic metrics, including taxa ratios, enterotypes, and metabolotypes. These were then compared with phenotypic data, with very few studies also reporting raw unadjusted analyses. These findings were also not comparable to other research. Similarly, α-diversity metrics were not calculated on the same type of data and therefore not generalizable. Finally, none of the studies reporting SCFA concentration were compared to age, so it is unclear if they vary with age.

### 4.6. Strengths and Limitations of This Review

Strengths of this review include the broad initial search parameters and consideration of both geographical regions and age groups. The review emphasizes the importance of primer selection and highlights the necessary caution needed when comparing sequence data obtained by analyzing different regions of the *16S rRNA* gene. One limitation when comparing data using different workflows and databases is that pipelines may produce different results limiting the generalizability. An additional limitation of this study is that results were not separated by factors that could potentially influence community structure within populations. This includes fecal collection methodology and participant characteristics, such as body composition. Therefore, our results need to be interpreted with caution and may not be generalizable to different populations. A standardized workflow in the future would allow high-level individual age-related characteristics to be explored. However, collection method and analysis of samples would still be a factor.

### 4.7. Consideration for Future Research

Future studies should consider analyzing the functional capacity of their participants gut microbiome. Ideally by inclusion of metabolomics but alternatively consider a metagenomics approach or use resources that can infer metabolic function, such as the bioinformatics software tool, Phylogenetic Investigation of Communities by Reconstruction of Unobserved States (PICRUSt) [118,119]. This would start to progress research beyond associations and allow causative links to be explored [120]. More consideration and measurement of confounders, such as diet, needs to be collected, and more research in low- and middle-income countries is needed to help elucidate and validate differences between and within geographic locations. Going beyond the analysis of the bacterial portion of the gut microbiome will also help define the true composition of the preadolescent gut microbiome.

## 5. Conclusions

In conclusion, the preadolescent gut microbiome of children was dominated by Firmicutes and Bacteroidetes, like the adult gut. Geographic location, age and *16S rRNA* region are associated with specific taxonomic characteristics emphasizing the importance of comparing studies from similar geographic regions, and settings within this region, at similar ages using similar primers. There were limitations in the way raw sequence data was processed, including database used for read classification, presenting OTUs at different ranks, limiting the ability to compare between studies. This review also highlighted the need for robust, well validated methods for analysis of SCFA. Future research with larger studies and more phenotypic data are required to better understand the development and composition of the pediatric gut and its importance for the future health of the child.

## Figures and Tables

**Table 1:** Participant characteristics in included studies.

First Author Year [REF]	Cohort Location	Study Population	Study Design	Age Range	Number of Participants	*16S rRNA* Target Region	Sequencing Platform	Summary
Avershina 2014 [63]	Norway	Healthy infants (IMPACT Cohort)	Longitudinal	2 years	39	V3-V4	454	The butyrate producing family *Lachnospiraceae* dominated the microbiome of the two-year-old children (46%). Followed by unclassified Actinobacteria and *Faecalibacterium*.
Berding 2018 [64]	USA	Healthy preadolescents	Longitudinal	4–8 years	22	V3-V4	MiSeq	Baseline dietary patterns are associated with temporal stability of microbiota over a 6-month period
Bisanz 2014 [65]	Tanzania	Healthy children exposed to heavy metals	Longitudinal	6–10 years	40	V6	Ion torrent	Probiotic yoghurt did not significantly impact community composition. Gut was dominated by *Prevotellaceae*, similar to the other African study (De Filippo et al. [31]).
Ringel-Kulka 2013 [66]	USA	Healthy children	Cross-sectional	1–4 years	28	V1 and V6	HITChip Microarray	Firmicutes dominated the gut microbiome at a phylum rank. *Clostridium cluster XIVa* was the most abundant taxon. Authors suggest microbiota is not adult-like at 4 years.
Cheng 2016 [28]	USA	Healthy children	Longitudinal	1–5 years	28	As above	As above	Bacterial diversity did not increase with age. All age groups were dominated by Firmicutes at the phylum. At genus rank, it was *Ruminococcus*, *Clostridium* and *Bifidobacterium*. The authors conclude that at age five the microbiome is still developing towards a stable adult-like composition.
De Filippo 2010 [31]	Italy (EU), Burkina Faso (BF)	Healthy children	Cross-sectional	1–6 years	29	V5-V6	454	Significant difference in community structure. BF gut dominated by genera *Prevotella* and *Xylanibacter*. EU gut dominated by genera *Faecalibacterium* and *Bacteroides*.
De Filippo 2017 [67]	As above	As above	Cross-sectional	2–8 years	37	As above	As above	Burkina Faso rural children had a gut dominated by bacteria of the *Prevotellaceae* family (66.8%). This trended downwards as the participants became less rural and was even more extreme for the Italian children, where the relative abundance of *Prevotellaceae* was only 0.44%.
Schloss 2014 [68]	USA	Healthy children	Longitudinal	2–10 years	4	V3-V5; WGS	454	As others have concluded, intra-individual variability is less than inter-individual microbiome variability.
Ghosh 2014 [69]	India	Data of healthy children only	Cross-sectional	2.5–6 years	5	WGS only	454	Apparently healthy Indian children’s gut microbiome were dominated at the phylum rank by Bacteroidetes and Firmicutes and at the genus rank by *Prevotella*. This is similar to those in other developing countries, such as Burkina Faso [31] and Tanzania [65]
Hollister 2015 [70]	USA	Healthy preadolescents	Cross-sectional	7–12 years	37	V3-V5; WGS	HiSeq 2000	In *16S rRNA* analysis the gut microbiome was dominated at the genus rank by *Bacteroides*, *Faecalibacterium* and *Alistipes*. In the WGS subgroup analysis, *Bacteroides*, *Faecalibacterium*, *Bifidobacterium* and *Alistipes* dominated at the genus rank.
Jakobsson 2014 [19]	Sweden	Healthy infants	Longitudinal	2 years	24	V3-V4	454	At two years old, the gut microbiome was dominated at the phylum rank by Firmicutes and at the genus rank by uncl. *Lachnospiraceae*, *Bacteroides* and *Bifidobacterium*. Similar to results from Avershina et al. [63].
Chong 2015 [71]	Malaysia	Healthy children	Cross-section	7–12 years	61	V3-V5	454	Despite differences in hygiene practices and socioeconomic status, Chinese and Malays were not significantly different. Orang Asli were significantly different to other ethnicities.
Kisuse 2018 [32]	Thailand	Healthy children	Cross-sectional	9–11 years	45	V1-V2	MiSeq	Rural children followed a more traditional plant-based diet and had higher SCFA production and a functional gut reflecting this. City of residence significantly associated with community structure even after adjustment for age and gender. At phylum rank, the microbiome was dominated by Bacteroidetes and Firmicutes. At the genera rank, *Bacteroides*, *Prevotella* and *Faecalibacterium* were the most dominant.
Koenig 2011 [25]	USA	Healthy child	Longitudinal	1–2.5	1	V1-V2	454	Bacteroidetes and Firmicutes dominate the child’s microbiome at from one year onwards. This is associated with an increase in SCFAs and an enrichment of carbohydrate utilization genes. Results suggest that the 2.5-y-old human gut microbiome has many of the functional attributes of the adult microbiome.
Lim 2015 [24]	USA	Healthy children	Longitudinal	2 years	8	V4	MiSeq	24-month microbiome was dominated at the family rank by *Lachnospiraceae* and *Ruminococcaceae*. Both of which contain known butyrate producers.
Lin 2013 [72]	Bangladesh, USA	Healthy children	Longitudinal	9–14 years	10	V1-V3	454	*Prevotella* and *lactobacillus* were higher in NE children, as was vegetables. Both of which were correlated. *Prevotella* was higher in others following a traditional plant-based diet [31,32,65].
López-Contreras 2018 [73]	Mexico	Healthy children	Cross-sectional	6–12 years	138	V4	MiSeq	Both microbiomes were dominated at the phylum rank by Bacteroidetes (67.5% in normal weight children and 69.4% in obese children) and Firmicutes (27.8% in normal weight and 26% in obese children). At the genus rank, the four most abundant bacteria were *Bacteroides* (39.0%), *Prevotella* (24.0%), unclassified *Lachnospiraceae* (7.2%) and unclassified *Ruminococcaceae* (6.1%).
Nakayama 2015 [74]	China, Taiwan, Japan, Indonesia, Thailand	Healthy children	Cross-sectional	7–11 years	303	V6-V8	454	Overall, at the phylum rank the microbiome was dominated by Firmicutes (61.98%) and at the genus rank by *Bifidobacterium*, *Faecalibacterium* and *Bacteroides* (overall average above 10% each). Two Indonesian cities show highest α-diversity and Japanese cities reported lowest α-diversity.
Nakayama 2017 [75]	Philippines	Healthy preadolescents	Cross-sectional	7–9 years	43	V6-V8	454	Baybay children’s microbiome was dominated by the family *Prevotellaceae*, whose diets had significantly less fat. Ormoc children by the families *Ruminococcaceae* and *Lachnospiraceae*.
Nicolucci 2017 [76]	Canada	Overweight or obese children (>85th BMI percentile)	Longitudinal	7–12 years	42	V3	MiSeq	At a phylum rank, microbiomes were dominated by Firmicutes (prebiotics group = 68.6%, placebo group = 68%) and Bacteroidetes (both 14.7%).
Riva 2017 [77]	Italy	Healthy children	Cross-sectional	9–16 years	78	V3-V4	MiSeq	At all three taxa ranks there was a clear distinction in microbiota composition between normal weight and obese children. F:B ratio was significantly higher in obese children. This is similar to other studies examining body composition and F:B ratio ([78,79]).
Smith-Brown 2016 [80]	Australia	Healthy preadolescents	Cross-sectional	2–3 years	37	V6-V8	MiSeq	Correlations and UniFrac analyses indicated that intake of several food groups is associated with various genera and microbial composition.
Smith-Brown 2018 [81]	As above	As above	As above	As above	As above	As above	As above	Weighted UniFrac is associated with FFMI z-scores in all participants but only significant in boys (when stratified by gender).
Hollister 2018 [82]	USA (Hispanic children)	Obese children	Longitudinal	2–5 years	52	V1-V3	MiSeq	Despite significant weight loss in the intervention group, limited shifts in community composition were seen, including no significant changes in α-diversity.
Yassour 2016 [13]	Finland	Healthy infants (DIABIMMUNE study)	Longitudinal	2–3 years	39	V4	16S: MiSeq; WGS: HiSeq	Antibiotic positive children had reported greater instability between consecutive samples. The microbiome of the whole group was dominated at the family rank at 24 to 36 months by *Bacteroidaceae* (42%), *Ruminococcaceae* (17%) and *Lachnospiraceae* (14%).
Zhou 2016 [83]	China	Healthy twins	Cross-sectional	1–6 years	14	WGS	HiSeq 2500	The genus *Bacteroides* dominated the gut (36.6%), followed by *Eubacterium* (11.1%) and *Bifidobacterium* (6.98%).
Murugesan 2015 [84]	Mexico	Healthy children	Cross-sectional	9–11 years	190	V3	Ion torrent	Normal weight and obese children had a similar relative abundance of Actinobacteria, Bacteroidetes and Firmicutes, however, there were 2.5 times more relative Proteobacteria in normal weight children.
Monira 2011 [85]	Bangladesh	Data of healthy children only	Cross-sectional	2–3 years	7	V5-V6	454	*Prevotella* and *Bacteroides* dominated the microbiome of healthy Bangladeshi children.

Sequencing platform: 454 = Roche 454 pyrosequencer; MiSeq = Illumina MiSeq; HiSeq = Illumina HiSeq. SCFA: short chain fatty acid; WGS: whole genome sequencing. Additional information: for detailed age (study means were taken where group means were not available), participants in each group, see Table S12.

## Supplementary Materials

The following are available online at https://www.mdpi.com/2072-6643/12/1/16/s1, Figure S1. PRISMA Flowchart and Checklist, Figure S2. Family by location of cohort, Figure S3. Family by age, Figure S4. Family by *16S rRNA* region sequenced, Figure S5. Genus by location of cohort, Figure S6. Genus by age, Figure S7. Genus by *16S rRNA* region sequenced; Table S1. Phylum by location of cohort, Table S2. Phylum by age, Table S3. Phylum by *16S rRNA* region sequenced, Table S4. Family by location of cohort, Table S5. Family by age, Table S6. Family by *16S rRNA* region sequenced, Table S7. Genus by location of cohort, Table S8. Genus by age, Table S9. Genus by *16S rRNA* region sequenced, Table S10. Alpha diversity, Table S11. Short chain fatty acid concentrations, Table S12. Detailed study information, dietary and other analysis.
